# Supplementary material for: Are carbon emissions trading and green financial instruments synergistic? -Comprehensive quantitative research based on content analysis
Source: PLoS One. 2024 Mar 7;19(3):e0298601. doi: 10.1371/journal.pone.0298601 (PMC10919601; doi:10.1371/journal.pone.0298601)
Supplement: S1 Data — (ZIP) [file pone.0298601.s001.zip › Data/地级市Z_F工作报告.zip!/╣π╢1⁄2/╓Θ║ú╩╨/└┤╫╘╣1⁄2╓┌║┼í╛┬φ┐╦╩2╛▌═°í┐_2003.docx]

各位代表：

　　我代表市人民政府向大会作政府工作报告，请予审议，并请政协委员和其他列席人员提出意见。

　　一、2002年的工作回顾

　　2002年是珠海继续加快发展的一年。市政府在省委、省政府和市委的领导下，在市人大、市政协监督支持下，高举邓小平理论伟大旗帜，努力实践“三个代表”重要思想，认真贯彻省第九次党代会精神，着力构筑实业旺市、科教兴市、环境强市三大发展平台，扩大对内对外开放，妥善处理改革、发展和稳定的关系，全市国民经济持续健康发展，精神文明建设和社会各项事业发展成效显著，人民生活水平稳步提高，较好地完成了市五届人大四次会议确定的任务。

　　（一）国民经济保持良好发展势头。

　　全年实现国内生产总值410.6亿元，比上年增长12.4％。其中，第一产业增加值16.2亿元，增长7.4％；第二产业增加值225.2亿元，增长12％；第三产业增加值169.2亿元，增长13.5%。三次产业比重由上年的4.1：55.3：40.6调整为3.9：54.9：41.2。全市财政总收入44.3亿元，其中，财政一般预算收入按新口径为31.2亿元，增长13.1％。进出口总额128.4亿美元，增长31％。固定资产投资总额120.4亿元，增长14.8％。按新口径，城镇居民人均年可支配收入15320元，扣除物价因素，实际增长8.4％。农渔民人均年纯收入5097元，增长6.2％。

　　农业和农村经济结构调整加快。农业总产值26.5亿元，增长6.9％。水产养殖面积扩大到32万亩，养殖品种结构进一步优化。白藤湖现代农业公司、北极品水产等一批农业龙头企业发展壮大。引导农村青年到工厂就业，降低农村电价，农民负担减轻。

　　工业展现喜人前景。工业总产值790.8亿元，增长12.3％。PTA、MTU、九州松下、三井铜箔等一批新建项目陆续建成投产，佳能、伟创力、三美、格力等一批骨干企业增资扩产或进行技术改造，一批中小企业快速成长。

　　开放型经济发展迅速。招商引资成效显著，新引进外商直接投资项目704个，合同利用外商直接投资13.2亿美元，增长87.7%；实际利用外商直接投资6.98亿美元，增长34％。出口总额52亿美元，增长37.4％。对内开放取得新成绩，与南方航空等大型企业扩大合作，吸引了一批国内优势企业到珠海投资。

　　服务业和物流业显著扩大。吸引了香港中旅投资开发珠海温泉。旅游接待人数突破1000万人次，其中过夜游客494万人次，增长15.2％；旅游总收入91.3亿元，增长16.2％。房地产开发档次逐步提高，居民二次置业成为热点，商品房销售面积134.1万平方米，增长12.2％。商贸市场兴旺，社会消费品零售总额143.5亿元，增长11.7％。保税区仓储和物流业增长较快，珠海港成为全省最大的液化气储运基地。全市港口货物吞吐量2240万吨，增长13％；机场客、货吞吐量分别增长14.6％和15.8％。

　　金融财税平稳运行。“金融安全区”建设取得成效，金融机构资产质量提高。国税、地税部门优化服务，加强征管，税收收入稳步增长。财政资金、行政性收费和罚没收入管理进一步规范，进行国库集中支付试点，财政资金使用效益提高。

　　区域经济活力增强。实施“园镇互动、强镇富民”重要决策，园区和镇级经济发展形成合力。香洲区现代服务业快速发展，经济发展质量提高。斗门区、金湾区工业化和农业产业化取得新进展，各项经济指标大幅增长。横琴经济开发区、万山海洋开发试验区旅游业兴旺，水产业渐成特色。

　　（二）改革开放迈出新步伐。

　　机构和干部人事制度改革稳步推进。区级行政机构和全市事业单位改革全面展开。市直事业单位从270个减少到166个，人员编制精简了53.7％。改革事业单位经费运行机制，变“以费养人”为“以费养事”。面向社会公开招考行政机关工作人员，又有一批优秀年轻干部通过公开选拔走上领导岗位。

　　国有企业和国有资产管理体制改革成效显著。国有经济结构调整取得新进展，又有60家劣势企业实现国有资本退出。管资产和管人、管事相结合的国有资产三级经营管理架构运行良好，国有资产运营效益逐步提高，市属国有资产保值率连续第三年达到105％。

　　民营经济发展势头良好。全市私营企业和个体户发展到42766家，其中私营企业6539家，增长12.8％。民营资本成为承接国有资本退出和转让的主要力量，民营科技企业异军突起，在房地产、商贸流通等领域，民营企业都发挥着主力军作用。

　　市场配置资源水平提高。政府采购和有形建筑市场运用信息化手段，实现“全程监控”和“阳光操作”。按规范化要求新设立了商用土地交易中心。市产权交易中心被省确定为区域性产权交易中心。对政府投资建设工程实行集中监管，提请市人大审议通过《珠海市政府投资项目管理条例》，规范了政府投资行为。学生装实行招标采购，逐步推行中小学校后勤管理社会化。行业协会等中介组织发展加快。

　　整顿市场经济秩序成效明显。开展集贸市场、加油站、职业中介以及旅游、外汇、建筑和房地产市场专项整治，制假售假、走私贩私、偷税骗税、逃汇骗汇、非法传销等违法行为受到惩处，社会信用水平逐步提高。公开销毁大批盗版光碟，行政机关带头使用正版软件。安全生产管理得到加强。

　　社会保障体系更加健全。制订和落实了促进再就业政策，城镇登记失业率为2.91％。社保扩面征缴又有新突破，社保基金收支状况良好，结余稳步增长。“两个确保”全面落实，大病医疗保险受到广大外来工欢迎。实施《珠海市企业工资支付条例》，企业工资支付行为逐步规范。

　　对外开放水平提高。清理、修订和废止了一批与世贸组织规则不一致的政府文件。在全市政权机关全面推行政务公开。成功举办第四届中国国际航展、第五届粤台经贸交流会和首届旅外乡亲恳亲大会。积极配合澳门新边检大楼建设，取消横琴大桥收费，横琴口岸延长通关时间。电子口岸建设加快，九洲口岸试行无纸通关。全年经我市口岸出入境旅客5370万人次，增长18.5%。优势企业“走出去”获得初步成功，格力空调巴西生产厂投产。

　　（三）科教兴市成效显著。

　　科技创新环境不断优化。积极创建全国科技进步先进市。加大对科技企业的扶持力度，市级科技三项经费增加到8200万元。国家级软件产业基地、国家级农业高新技术示范园区、省高科技成果产业化示范基地、民营科技园、留学人员创业园等科技园区建设如火如荼，形成多层次科技研发和示范体系。一批企业孵化器快速发展，哈工大新经济资源开发港运作良好，清华科技园投入使用，南方软件园二期加紧建设。

　　国家高新区和高新技术产业蓬勃发展。各科技园区、科技创新海岸基础设施建设加快，招商引资势头良好，高新区工业总产值188亿元，增长76％。全市新增经省认定的高新技术企业14家，总数达到79家。全市高新技术产品产值213亿元，增长15％，占工业总产值的26.9％。软件产品产值23亿元，增长44％。金山软件在国内政府采购中屡次中标。新增国家级火炬计划项目8个、星火计划项目1个、重点新产品计划项目4个。新增智能小家电和智能卡两家省级工程技术中心。全市专利申请量825件，增长10％。

　　创建全省信息化建设综合试点城市。编制完成“十五”信息化规划和集成电路产业发展规划。改造政府网站，初步建成电子政务平台。国际信息检索中心启用，企业信息化和社会信息化水平提高。互联网用户达19.5万户，增长10.8％。

　　大学园区有新发展。中大珠海校区、暨大珠海学院扩大招生规模，北京师范大学、北京理工大学、遵义医学院和省科技干部学院的珠海校区首期建成开学，一批产学研项目投入运作，吉林大学珠海校区开工建设，新引进中国人民大学、中央戏剧学院、中央音乐学院、北京化工大学、沈阳药科大学、中国医科大学等著名院校前来办学或兴办产学研基地。全市全日制在校大学生超过2万人，珠海大学城正发展成为广东省高等教育的重要组成部分。

　　基础教育和职业教育得到加强。以区为主的义务教育管理体制全面落实，基础教育投入大幅增加，优质教育资源明显扩大，薄弱学校改造加快，规范化小学陆续建成开学，全面启动创建国家级示范性普通高中工程。提升职业教育水平，筹建城市职业技术学院，与企业合作开展职业培训。民办教育有序发展。

　　（四）城市规划建设管理水平提高。

　　城市规划取得重要成果。新修编的城市总体规划经省政府批准，已上报国务院审批。完成唐家湾、南湾、高栏石化新城、金湾中心城区和“两岛一路”等详细规划以及城市交通、消防、运输枢纽规划等多项专项规划。工业园区和中心镇规划有序展开。

　　城市化迈出新步子。城中旧村改造稳步推进，30个自然村确定了开发商，22个自然村动工改造，41.3万平方米回迁房交付使用。加快镇村重组，撤并了6个镇，湾仔、前山撤镇改为街道办。

　　基础设施不断完善。昌盛大桥、横坑大桥、金唐路动工兴建，完成珠海大道部分桥梁、农村地方道路危桥改造，建设京珠高速公路珠海段西侧辅道。水务一体化优势逐步显现，整合供水资源，供水保障能力增强。加快电网改造，城乡用电实现同网同价，斗门220千伏变电站等工程建成启用，供电可靠性进一步提高。

　　城市管理水平提高。提请市人大审议通过《珠海经济特区市容和环境卫生管理条例》，城市管理有法可依。开通“城管110”，建立城市管理行政执法快速反应机制。市属公园全部免费对公众开放，部分单位文体设施等资源对社区开放。香洲区成为首批“全国社区建设示范区”。成功引入华电集团和法国威望迪公司运营、建设城市污水处理厂。积极尝试户外广告、洗车场等公共资源市场化经营。

　　社会治安管理进一步加强。深入开展严打整治斗争，加强社会治安综合治理，严厉打击各种刑事犯罪活动，改革警务管理体制，市民安全感进一步增强。

　　生态环境质量提升。编制完成生态城市建设规划纲要。建立起区镇环保目标考核责任制，环保“一控双达标”成果得到巩固，城市环境综合考核成绩全省第一。拱北污水处理厂三期工程竣工，城市生活污水集中处理率提高到60％以上。启动“生态家园”十年植树工程，建设“城市森林”。

　　（五）社会各项事业和精神文明建设取得新进展。

　　民主法制和政府自身建设得到加强。坚持向市人大及其常委会报告工作、向市政协通报情况。认真配合市人大做好立法工作。切实办好整合医疗卫生资源等市人大议案和一大批代表建议及政协提案。政府决策认真听取民主党派、工商联、无党派人士和专家小组的意见，建立起领导接待群众来访日制度。以建设服务型政府为目标的机关作风建设深入开展，窗口服务和现场服务走上制度化轨道。普法、行政复议和仲裁等工作得到加强。强化社会和新闻舆论对政府工作的监督。交通、物价、质监、旅游行风评议取得成效。开展廉政效能和行政效能监察，推行村账镇代管，从源头上预防和治理腐败，查处了一批违法违纪案件。

　　精神文明建设取得新成果。加强社会公德教育，普及科学知识，反对邪教，倡导健康文明的生活方式。社区和广场文化活动丰富多彩。建成镇级达标文化站等一批文化基础设施。新闻文化事业健康发展，广播电视覆盖率提高。网吧、歌舞厅等娱乐场所专项整治取得成效。提请市人大审议通过《珠海市见义勇为人员奖励与保障条例》，弘扬社会正气。

　　社会各项事业全面发展。群众性体育运动蓬勃开展，珠海运动员在省运会上取得好成绩。中山大学附属第五医院建成开业。深化医疗卫生体制改革，医疗卫生和保健水平提高。全面贯彻《人口与计划生育法》和《广东省人口与计划生育条例》，计生工作继续走在全省前列。市民国防意识增强，双拥共建成绩突出，征兵工作连续第22年实现全优。解决特困群众住房难、就医难、打官司难、子女入学难取得显著成效，对口扶持工作力度大、效果好。侨务、外事、对台、审计、监察和统计工作更好地为经济和社会发展服务。人防、档案、气象、民族、宗教、老年人、残疾人工作又有新发展。工会、共青团、妇联、科协、红十字会、工商联、文联、侨联、社科联和港澳流动渔民工作都有新进步。

　　（六）办理十件实事取得实效。

　　一是在十六大召开前实现村村通有线电视。

　　二是建设地理信息系统工程，国土房产登记交易信息管理系统试运行，制定了数据共享标准，数据采集等工作正加紧进行。

　　三是乾务、六乡、平沙、小林四所规范化小学如期建成开学，淇澳兆征学校教学楼建成启用。

　　四是珠海报业大厦竣工并投入使用。

　　五是完成斗门区自来水改造工程，斗门区农村全部用上达标自来水。

　　六是梅溪路建成通车，旅游景区形象和环境大大改善。

　　七是吉柠路建成通车。

　　八是修通了夏湾、新香洲和前山地区14条烂尾路。

　　九是万山港口岸对外开放已通过国家正式验收。

　　十是莲花路商业步行街区改造牵动该片区总体功能优化，改造方案已广泛征求专家和市民意见，目前正按市场化方式加紧推进。

　　各位代表，2002年政府工作取得的这些成绩来之不易，这是省委、省政府和市委正确领导的结果，是与中央、省驻珠单位和驻珠军警部队的突出贡献分不开的，是全市人民努力工作和海内外友人大力支持的结果。在此，我谨代表市人民政府向全市人民和海内外各界朋友表示衷心的感谢！

　　我们也清醒地认识到，我市经济和社会发展中仍存在一些突出问题。主要是：工业化进程相对较迟缓，技术创新和产业配套能力不强；经济总量不大，区域经济发展不平衡，镇村经济薄弱；民营经济发展有不少差距，国有经济布局尚有待优化；基础设施不够配套，海港、空港未能充分发挥应有作用；原有的一些比较优势有所弱化，综合竞争力和发展后劲亟待增强等。这些问题需要我们在发展中予以高度重视，并着力加以解决。

　　二、2003年的工作安排

　　今年是贯彻落实十六大精神全面建设小康社会的第一年。我们要进一步增强责任感和紧迫感，深入学习贯彻十六大精神，用十六大精神统一思想、鼓舞士气、凝聚力量。我们必须坚持解放思想、实事求是、与时俱进，务必保持谦虚谨慎、不骄不躁的作风，务必保持艰苦奋斗的作风，紧紧抓住发展这个执政兴国的第一要务，始终保持昂扬向上的精神状态；我们必须正确认识自我，勇于承认差距，善于扬长补短，尊重客观规律，聚精会神搞建设，一心一意谋发展。

　　今年市政府工作的总体要求是：高举邓小平理论伟大旗帜，全面贯彻“三个代表”重要思想，认真落实党的十六大精神，以“增创新优势，更上一层楼，率先基本实现社会主义现代化”为总目标总任务统揽工作全局，按照中央经济工作会议、省第九次党代会、省委九届二次全会和市委四届七次全会的部署，坚持实施外向带动、科教兴市、可持续发展和区域协调发展战略，突出抓好制度创新、科技创新和扩大开放，加快推进新型工业化，加快推进城市化，加快推进国民经济和社会信息化，继续加强基础教育和农业、农村工作，继续优化投资发展环境和生活环境，不断加强政府自身建设，以扎扎实实的工作，努力增强珠海的综合实力、城市竞争力和发展后劲，确保十六大后第一年的工作开好头、起好步，向全面建设小康社会和“三基地一中心”的发展目标迈出坚实的步伐。

　　今年我市经济发展的主要预期目标是：国内生产总值比上年增长11％，工业总产值增长13％，农业总产值增长5％，固定资产投资增长10％，外贸出口额增长9％，实际利用外商直接投资增长8％，财政一般预算收入增长11％，社会消费品零售总额增长11％，城镇居民人均年可支配收入增长5％，农渔民人均年纯收入增长6％。

　　今年政府工作的重点是：

　　（一）全力推进新型工业化，增大经济总量，提高经济质量。

　　认真贯彻实施《中共珠海市委关于大力推进新型工业化的决定》，是当前经济工作中最紧迫的任务。

　　营造大办工业的环境和氛围。政府各职能部门都要围绕工业发展，进一步解放思想，转变观念，简政放权，强化服务。要将政府可支配资源和政府服务向大办工业倾斜，尽快增大工业经济总量，提升工业经济质量，争取走出一条科技含量高、经济效益好、资源消耗低、环境污染少、人力资源优势得到充分发挥的新型工业化路子。

　　继续实施功能区带动战略和“园镇互动、强镇富民”举措。进一步增强经济功能区的功能优势和产业特色，加快高新区、临港工业区、保税区基础设施建设，增强项目承载能力。按照“统一规划、滚动发展、集约建设”的原则，合理安排开发时序，提高园区土地利用效率和投资效益。

　　扩大工业发展载体。向国务院申报扩大保税区面积。加快新设的龙山、富山、三村、平沙、联港五个工业区的开发建设，鼓励镇级工业园和工业小区的发展，形成市、区、镇三级工业园区优势互补、互动发展的格局。市重点吸引高新技术和龙头项目，区、镇工业园区放手发展配套项目和一般中小项目。在符合规划和环保的前提下，让资金技术密集型项目与劳动密集型项目、大型项目与中小型项目都能找到合适的发展空间。

　　创新园区开发运作模式。积极运用市场机制，充分调动基层的积极性，广泛吸引外资、民间资本、金融资本参与园区开发，鼓励镇村以多种方式入股。规划和控制好园区周边的商用土地，加快生活配套设施建设，合理经营园区公共资源，形成投资─回报─再投资的良性机制。

　　培育优势产业和骨干企业。要重点围绕电子及通信设备制造业、电气机械及器材制造业、医药及医疗器械制造业、计算机软件业、石化原料及制品业等开展卓有成效的产业招商，增强产业实力，延长产业链。特别是要推进临港石化基地建设，争取在发展石化产业方面有更大突破。培育优势产业关键在于引进和培育龙头企业。要高度重视引进拥有知名品牌或自主知识产权、主业突出、核心竞争力强的龙头项目。同时，要高度重视帮助现有企业解决生产经营中的困难和问题，鼓励其增资扩产、做大做强。从今年起，市财政要增加技改贴息资金，支持企业技术创新和技术改造。实施名牌带动战略，鼓励企业创名牌，对新创全国名牌的企业要给予奖励。

　　大力扶持中小企业发展。贯彻落实《中小企业促进法》，研究和出台促进中小企业发展的具体措施，建立健全中小企业服务体系，引入信用联保机制，积极解决中小企业融资难问题。定期组织产业配套招商和专项配套洽谈活动，积极引导中小企业与大型企业产业协作配套。鼓励中小企业进行技术创新，向“专、精、特、新”方向发展，增强中小企业竞争力。

　　以信息化带动工业化。建立企业信息化协调机制，鼓励建立行业共性技术支撑服务平台，推广信息技术运用，提高工业设计、生产的自动化、智能化和管理信息化水平，降低物流、营销和管理成本，提升企业竞争力。积极运用信息技术和其它高新技术改造传统产业。

　　促进工业与现代服务业协调发展。依托工业发展，带旺人气和商气。进一步搞活商贸流通业，加快莲花路、香埠路商业步行街改造和特色商业街建设，积极支持新型商业业态发展。高度重视物流业发展，积极参与珠三角现代物流体系建设，机场、港口、保税区等重点区域要在规划上为物流业预留足够的发展空间。要把旅游业这一优势产业做大做强，大力发展休闲度假、商务和会展旅游，整合旅游文化资源，争取旅游项目招商有新突破。抓住我市作为开放金融业试点城市的契机，加快金融业发展。积极发展会计、审计、法律服务、信息咨询等中介服务业。

　　（二）以增加农民收入为中心，加快推进农业产业化，全面繁荣农村经济。

　　要认真贯彻“多予、少取、放活”的方针，加快推进农村改革和农村经济结构调整，提升农业竞争力，千方百计增加农民收入。

　　积极推进农业产业化经营。引导农民以市场为导向，扩大水产、蔬菜、水果、花卉和畜牧业规模。发展生态农业。扶持发展农产品加工业。加大农业招商引资力度，大力引进和培育农业龙头企业。做好国家级农业高新技术示范园区、省海洋科技园、白蕉省级现代农业示范基地、市水产种苗基地、水产养殖基地、无公害蔬菜基地和奶牛饲养基地等一批农业龙头项目的服务工作。

　　改善农村生产生活条件。加强水利等农业基础设施建设，规划迁建大型渔港，加快农村公路硬底化改造。建立健全农产品质量监督检测体系，逐步推行农产品质量安全市场准入制度。促进农业科技进步和技术推广，加强农民职业技能培训，推广良种良法。改善农村金融服务。推进农产品批发市场建设，积极培育农产品流通等中介组织发展。

　　促进农村社会全面进步。扶持农村集体经济发展。引导农村富余劳动力转向第二、三产业就业。稳步推进农村税费改革，清理涉农收费，减轻农民负担，调动和保护好农民的生产积极性。加快农村教育、科技、卫生、文化等社会事业发展。

　　（三）着力加快新区建设，提高城市化水平，促进区域协调发展。

　　实施“城市西拓”，是实现区域协调发展的必由之路。今年重点要抓好规划、基础设施配套和工业发展，加快推进城市化进程。

　　提高规划和土地管理水平。坚持高标准做好城市规划。加强规划宣传，扩大公众参与，提高规划法制化水平。要认真组织实施经国务院批准的城市总体规划，并按国务院要求，在上半年完成珠海市近期发展建设规划。加紧编制和深化重点发展区域分区规划、详细规划和市政规划。要十分珍惜宝贵的土地资源，制定基准地价，建立土地储备制度，规范土地出让，妥善处理闲置土地，进行土地盘整，有效整合土地资源，提高政府对土地资源的调控能力，提升土地价值。

　　加紧完善交通等基础设施。要抢抓机遇，争取各方面的支持，加快交通路网建设，为加快城市化和工业化创造基础设施条件。重点是完善珠海港和珠海机场集疏运系统，有效发挥“两港”的龙头作用。首先要建设珠港大道，同时积极推动粤西沿海高速公路珠海段、江珠高速公路、广珠西线高速公路等“三纵两横”高等级公路建设，使珠海更紧密地连接到珠三角高速路网。要积极推进珠海港5万吨级集装箱码头和大吨位石化码头建设，推动广珠铁路、广州至珠海城际快速轨道建设，做好珠港澳大桥等大型交通项目的相关工作。抓紧西部地区海堤建设，提高防洪保障能力。

　　加快斗门、金湾两个新区的城市化进程。城市化的动力在于加快工业化。今年要从规划和加快工业发展入手，启动井岸─白蕉新城、金湾─三灶新城、临港工业新城和龙山、三村、富山工业区组成的西部工业新城这四个新城组团的建设，推动工业园区与城镇互动发展。新区发展要走综合开发的路子，充分发挥现有条件，促进新型工业与现代农业、旅游业、商贸物流业、科技教育和房地产业等全面发展。新区建设要整合资源，积极推进镇村重组，加快中心镇建设步伐。加快新区城市化需要体制创新，城镇基础设施建设要向多元化投资主体开放，推进户籍制度改革，消除农民向城镇迁移的障碍。

　　香洲主城区要优化提高。加强房地产业宏观调控，在稳定城中旧村改造相关政策的基础上，结合市场需求和城市规划，有计划、有步骤地逐步放开城中旧村以外的房地产开发项目，确保房地产业有序发展。积极改造主城区老工业区，推动产业升级。加快建设和完善城市客运枢纽站等公共配套设施。积极推进城市管道燃气项目建设，争取年内实现部分片区供气。进一步提高城市管理水平，积极开展城市经营，加强城市管理执法队伍建设，加大城市管理综合执法力度，大力整治市容市貌。要提升和优化主城区的综合环境，吸引高水平的教育、科研项目和国内外大型机构落户，加快现代服务业发展，推动经济结构优化升级。

　　主城区向南北新城组团拓展。以建设情侣北路为重点，加快唐家湾地区基础设施建设，依托大学园区和科技创新海岸，大力发展高等教育、高新技术产业和新兴服务业。要在年底前建成昌盛大桥，以此推动南屏、湾仔、洪湾、横琴新组团的开发建设，带动制造业和以物流业、休闲旅游业为龙头的现代服务业发展。

　　海岛开发要形成特色。要在保护好海洋资源和海岛生态环境的前提下，做好海岛综合开发利用规划，加快海洋经济发展。抓住万山港口岸对外开放的机遇，加快万山海洋开发试验区海上中转仓储、海洋旅游业和海洋渔业发展，走发展特色经济的路子。

　　（四）大力推动制度创新和扩大开放，为发展增添动力。

　　我们必须在制度创新和扩大开放方面有新招，出实招，才能充分发挥多元化市场主体的积极性，形成生机勃勃的发展局面。

　　继续深化国有企业和国有资产管理体制改革。以产权制度改革为重点，着力在引进战略投资者等方面有新突破，推进国有企业产权多元化。加快国有经济布局和结构调整，尽快完成重点板块国有企业的整合重组，在优势领域做强做大。进一步完善国有资产管理体制，建立有效的激励和约束机制，推行国有资产经营预算管理办法，确保国有资产保值增值。

　　大力发展民营经济。认真贯彻省委、省政府《关于加快民营经济发展的决定》，按照“政治平等、政策公平、法律保障、放手发展”的方针，尽快出台我市加快民营经济发展的政策措施。大力发展科技型、生产型、外向型民营企业，积极吸引内地的民营企业到珠海发展。依法保护民营企业的合法财产和权益。放宽市场准入条件，凡国家未作限制性规定以及加入世贸组织后对外资开放的行业和投资领域，都对民营企业开放。吸引民营资本以独资、参股、控股、联营等方式参与公共基础设施、公益事业项目的投资建设和运营管理，鼓励民营企业参与国有企业改组、改造，发展混合所有制经济。采取多种方式，积极解决民营企业融资难问题。引导民营企业技术创新和制度创新，建立现代企业制度，促其上规模、上水平、创名牌。

　　深化财税和投融资体制改革。加快完善公共财政框架，优化财政支出结构。全面推行部门综合预算和国库集中支付改革，科学理财，增收节支。规范政府资产管理，强化财政监督和预算执行审计。严格依法治税，加大力度培植税源。积极培育上市公司，扩大资本市场融资规模。加强金融监管和地方金融资产管理，防范和化解金融风险。进一步规范政府投资行为，着力启动民间投资，扩大投资规模。

　　努力完善市场体系。要在更大程度上发挥市场配置资源的基础性作用，发展资本、产权、土地、技术、人才、有形建筑等要素市场，加强市场监管，形成统一开放、竞争有序的市场体系。充分发挥已建立的产权交易中心、建设工程交易中心、商用土地交易中心、政府采购中心、人力资源开发管理服务中心、市财政国库支付中心和市政府投资建设工程管理中心等在资源配置中的作用，提高运作水平。深化流通体制改革，积极发展现代流通方式。

　　提高利用外资水平。加大对欧美日韩及港澳台等国家和地区的招商力度，积极吸引跨国公司和大财团的直接投资，着力吸引外商在我市设立地区总部、制造业基地以及研发中心。把利用外资与国有企业改组改造结合起来，鼓励和引导外资参与国企的股份制改造、技术改造。以服务贸易为重点，在金融、信息服务、旅游、会展、中介服务等领域扩大利用外资。

　　拓展国内国外两个市场。发挥口岸优势，加快“大通关”建设，提高通关效率。努力扩大出口，实施市场多元化战略，巩固传统市场，开拓俄罗斯、东欧、中东、南美、南非、印度等新兴市场。坚持科技兴贸，以质取胜，提高出口商品和服务的技术含量和附加值。鼓励和支持有条件的各种所有制企业“走出去”对外投资，建立销售网络、生产体系和融资渠道，开展各种经济技术合作。积极拓展内地市场，鼓励珠海企业参与西部大开发。

　　加强区域经济合作。主动寻求和扩大与珠江三角洲经济区域其它城市的经济合作，实现优势互补和互动发展。加强珠港澳台经济合作，扩大与周边城市在基础设施、服务、物流、旅游等领域的合作与交流。加快珠澳在建设跨境工业区、开发横琴岛等方面的合作步伐。积极推动珠海机场与周边城市的合作。

　　（五）努力推动科技创新和教育创新，提升城市核心竞争力。

　　城市竞争力要以人才和科技、教育实力作为支撑，要坚定不移地实施科教兴市战略，努力提升珠海的核心竞争力。

　　加快高新区和高新技术产业发展。依托高新区、科技创新海岸和大学园区的产业基地、产学研基地、孵化器等科技载体，着力引进和发展技术水平高、关联带动强、市场前景广的科技项目，继续巩固和发展壮大电子信息产业，培育发展生物制药、新材料、新能源等新兴产业。特别是要加大集成电路设计与制造和软件产业领域的招商力度，加快国家软件产业基地建设。加快建设地理信息系统和电子政务平台，以电子政务带动企业信息化和社会信息化。

　　优化科技创新环境。重点扶持建设一批高水平的专业化科技中介机构，逐步完善科技服务体系。加强知识产权保护。选择一批重大核心技术攻关和应用产业化项目，在政策、资金、采购和上市融资等方面给予重点扶持，支持大中型企业建立工程技术中心和技术开发中心，加快建立我市重点产业研发中心和博士后工作站。深化科技体制改革，用好、管好科技三项经费，试行部分科技计划项目特别是软课题研究项目公开招标。引进国内外风险投资机构，发挥风险投资的作用。

　　坚定不移地办好大学园区。尽快完善大学园区规划，合理确定办学规模、结构和区域布局。认真履行合作协议，加强大学园区的社会管理和服务，逐步提升已引进高校的办学层次和水平，积极引导大学的学科设置与我市以及珠三角地区重点发展的产业相结合，使高校不仅引得进，而且能发展好，充分发挥大学园区的综合效益，为珠海的经济社会发展提供人才和智力支持。

　　提高教育整体水平。启动香洲区建设教育强区工程，全面提升基础教育水平。合理配置教育资源，加大创建国家级示范性普通高中和等级学校力度，推动义务教育均衡化和高中教育优质化。将今年确定为教育质量年，加强教师培训，推进素质教育，普及英语和信息技术教育。发展职业教育和继续教育，积极培养应用型人才，加快城市职业技术学院建设。贯彻《民办教育促进法》，鼓励和规范社会力量办学。

　　（六）致力于优化经济发展环境，不断增强可持续发展能力和发展后劲。

　　环境是珠海的优势。我们要进一步珍视环境，优化环境，不断增强综合环境对资金和人才持续的吸引力，这是增强珠海发展后劲的着力点。

　　加大力度保护生态环境。高标准建设生态示范区。加强环境监测，实现重点污染源在线监控。推广清洁生产，开展环境污染专项治理。高度重视合理利用和调配水资源。加快洪湾港建设和前山港搬迁，整治前山河“一河两岸”。继续建设“生态家园”，打造“绿色珠海”城市品牌，申报省级红树林保护区，进一步提升生态环境质量。

　　优化经济发展环境。继续打击各种破坏市场经济秩序的违法犯罪行为，建立规范的市场经济秩序。加快信用体系建设，提高全社会信用水平。依法加大力度清理违规集资。切实落实国民待遇，对各种所有制企业一视同仁。进一步清理各种收费，减轻企业负担，降低投资综合成本，改善投资软环境。

　　维护良好的社会环境。继续整治社会治安秩序，坚持标本兼治，综合治理，完善打、防、控一体化的社会治安长效机制，维护社会稳定。全面贯彻《安全生产法》，坚持预防为主的方针，防止各种恶性事故、重大伤亡事故发生。市和区共同努力，采取多种措施，营造优美舒适的居住生活环境，提高居民生活质量。

　　营造干事创业的人文环境。尊重基层和群众的首创精神，充分调动和激发广大干部群众的积极性、主动性和创造性，努力形成上下齐心、政通人和的社会氛围。大力培养和引进各类人才，吸引更多的海内外高层次人才来珠海创业。重视专业技术人才队伍建设和人才资源整体开发，实行人才居住证制度，试行政府雇员制度，逐步完善人才激励机制，促进优秀人才脱颖而出。

　　（七）推动社会各项事业全面发展，进一步提高人民群众生活水平。

　　要着眼根本目的，切实维护、实现和发展最广大人民的根本利益，不断提高人民群众的生活水平和生活质量。

　　关心困难群体生活。继续采取有效措施加大扶贫帮困力度，帮助困难群众解决生产生活问题。加强法律援助工作。继续支持我市西部地区群众改善住房条件。加快金湾区供水系统改造，争取年内完成，提高西部地区供水保障水平。加快香洲区城郊的自来水系统改造。认真落实妇女儿童发展纲要。做好老龄人口和残疾人工作。

　　加强就业和社会保障工作。全面贯彻全国和全省再就业工作会议精神，进一步落实扶持再就业的政策措施，拓宽就业渠道，千方百计扩大就业。建立和完善市、区、街道（镇）三级劳动保障管理服务网络，依法推进覆盖全社会的社会保障制度，积极创造条件将我市欠发达地区城镇职工逐步纳入基本养老和基本医疗保险范围，完善特困职工医疗分类救助制度，落实失业保险和城镇居民最低生活保障制度，实现应保尽保。依法保护包括外来务工者在内的全体劳动者合法权益。

　　高度重视先进文化建设。弘扬特区人民敢为人先、勇于创新、务实进取的精神。推进文化建设和文化体制改革，积极发展社会科学、文学艺术、新闻出版、广播影视等文化事业和文化产业，促进文化与经济融合。积极保护和合理开发利用历史文化资源。大力发展社区、企业、校园等各类群众文化。以乡镇带村，实现先进文化“进村入户”。继续加强科普教育，弘扬科学精神。深入开展群众性精神文明创建活动。

　　发展社会各项事业。加快整合市人民医院和拱北医院，扩建市妇幼保健院，加强预防保健，提高医疗卫生服务水平。深化农村卫生改革，积极探索多种形式的农村医疗保障办法，改善农村医疗卫生条件。发展群众体育运动，办好第六届市运动会，大力发展体育产业。深入贯彻人口与计划生育法律法规，强化社区和流动人口计生工作，争创全国计生优质服务先进市。加强社区建设，拓展社区服务。重视和加强国防建设，落实优抚安置政策，巩固军政军民团结，争取第五次蝉联全国双拥模范城。继续认真做好人防、国安、外事、口岸、侨务、对台、民政、档案、气象、民族、宗教和港澳流动渔民工作，发挥好工会、共青团、妇联等群众团体的作用，推动社会各项事业健康发展。

　　（八）加强政府自身建设，提高工作效率和服务水平。

　　实现今年的政府工作目标，关键在于坚持依法行政，从严治政，提高政府自身的行政能力，努力推动政治文明建设。

　　加强民主法制建设。坚持和完善政府向人大及其常委会报告工作、向政协通报情况的制度，自觉接受监督。加强政府法制建设，深入开展法制宣传教育，推进政府运行法制化。强化执法监督，落实行政执法责任制和过错责任追究制度。切实办好人大议案、代表建议和政协提案。健全基层自治组织，完善村务公开和厂务公开。规范法律服务。加强行政复议和仲裁工作。

　　完善科学民主的决策机制。拓宽人民群众反映意见的渠道，建立社会公示和听证制度。全面实行政务公开，凡不涉及秘密的事项，政府机关作出的对社会公众具有普遍约束力的行政规定，事关全局的重大决策过程和出台的政策，以及群众普遍关心的事项等，都要以适当的形式向社会公开。推进决策科学化民主化，遵循客观规律和经济规律，严格按制度和程序办事，避免决策失误。

　　加快转变政府职能。政府集中精力做好经济调节、市场监管、社会管理和公共服务，减少和规范行政审批，继续加强窗口服务和现场服务，加强部门之间的协调和配合，形成合力，提高行政效能，节约行政成本。理顺市、区、镇三级政府事权关系。继续深化事业单位管理体制改革。

　　加强政风建设。全面落实“八个坚持、八个反对”，深入开展机关作风建设。加强公务员培训，要让公务员队伍成为创建学习型城市的主体力量之一。加强廉政建设和反腐败斗争，厉行节约，勤俭办事。加强监察和审计等工作，发挥舆论监督作用，努力建设一支政治坚定、业务精通、干净干事的公务员队伍。

　　（九）继续办好服务于广大人民群众的十件实事。

　　一是建设珠港大道并在年内实现通车。

　　二是实施再就业工程并取得阶段性的明显成效。全市“四类”下岗失业人员再就业率力争达到60％，失业人员就业率达到65％以上，城镇登记失业率控制在3％以内。

　　三是建成斗门区横山、五山和金湾区第一中心小学三所规范化小学。

　　四是年底前完成珠海图书馆建设并投入使用；同时，启动珠海妇女儿童活动中心和青少年科技文化活动中心的建设，明年中建成并投入使用。

　　五是新建市中心血站。

　　六是解决万山岛、东澳岛居民生活用水问题。

　　七是完成白蕉联围海堤除险加固达标首期工程。

　　八是建成社会保障信息系统。

　　九是完善城市污水管网配套建设。

　　十是改造一条城市道路。

　　全市各区、镇和街道办都要结合本区域的实际，扎扎实实地为群众办一批实事。

　　各位代表！今年政府的工作任务十分繁重，全体政府工作人员和全市人民要倍加团结，倍加求实，倍加奋进，在市委的领导下，沿着党的十六大为我们指明的前进方向，脚踏实地，扎实工作，以发展的新思路、改革的新突破、开放的新局面和各项工作的新举措，朝着全面建设小康社会和基本实现社会主义现代化的目标迈出更大的步伐！
